# Dispersal Behavior of *Tetranychus evansi* and *T. urticae* on Tomato at Several Spatial Scales and Densities: Implications for Integrated Pest Management

**DOI:** 10.1371/journal.pone.0095071

**Published:** 2014-04-17

**Authors:** Ginette Y. Azandémè-Hounmalon, Simon Fellous, Serge Kreiter, Komi K. M. Fiaboe, Sevgan Subramanian, Miriam Kungu, Thibaud Martin

**Affiliations:** 1 Cirad, UPR Hortsys, Montpellier, France; 2 *icipe*— Plant Health Department, Nairobi, Kenya; 3 INRA, UMR CBGP, Montpellier, France; 4 Montpellier SupAgro, UMR CBGP, Montpellier, France; Institute of Vegetables and Flowers, Chinese Academy of Agricultural Science, China

## Abstract

Studying distribution is necessary to understand and manage the dynamics of species with spatially structured populations. Here we studied the distribution in *Tetranychus evansi* and *T. urticae*, two mite pests of tomato, in the scope of evaluating factors that can influence the effectiveness of Integrated Pest Management strategies. We found greater positive density-dependent distribution with *T. evansi* than *T. urticae* when assayed on single, detached tomato leaves. Indeed, *T. evansi* distribution among leaflets increased with initial population density while it was high even at low *T. urticae* densities. Intensity and rate of damage to whole plants was higher with *T. evansi* than *T. urticae*. We further studied the circadian migration of *T. evansi* within plant. When *T. evansi* density was high the distribution behavior peaked between 8 am and 3 pm and between 8 pm and 3 am local time of Kenya. Over 24 h the total number of mites ascending and descending was always similar and close to the total population size. The gregarious behavior of *T. evansi* combined with its rapid population growth rate, may explain why few tomato plants can be severely damaged by *T. evansi* and how suddenly all the crop can be highly infested. However the localisation and elimination of the first infested plants damaged by *T. evansi* could reduce the risk of outbreaks in the entire crop. These findings suggest also that an acaricide treated net placed on the first infested plants could be very effective to control *T. evansi*. Moreover circadian migration would therefore accentuate the efficiency of an acaricide treated net covering the infested plants.

## Introduction

When a given environment becomes non-viable (e.g. food shortage or climatic constraints), individuals have to find better opportunities elsewhere and disperse [1], [2]. Dispersal is therefore considered a key factor to survival because it determines organism spatial distribution and population dynamics [3], [4]. The dispersal of spider mite has been investigated in several studies on tetranychids [5], [6], [7]. The mode of dispersal varies between species, stage of the life cycle, sex, external/social environments, and time [1], [8]. In the specie *Tetranychus urticae*, individuals live in huge groups, exhaust the host plant and must recurrently disperse to new host. Individuals of *T. urticae* can dispersed by active movement (i.e. by walking) [9], [10]; by phoresy (i.e. passive transport by another organism) [11], [12]; aerial displacement by air currents [13], [14]. The Silk threads produced by spider mites further participate to long-distance, aerial dispersal [15]. In *T. urticae*, a collective displacement occurs in conditions of overcrowding and food depletion: they form silk balls at the apex of their over-exploited hosts plants. These balls are characterized by phases of growth during the day and nocturnal size decrease. This is likely to be related to *T. urticae* circadian rhythm during which mite population move up in the early afternoon and migrate to the bottom of plants during the night [16]. The within plant distribution of mite may vary widely for different species of host plant and spider mite [17]. Many factors such as leaf surface, food availability and quality, leaf exploitation, predation, mite density, temperature, light and humidity can modify the within plant dispersal of mite. In the case of *Tetranychus evansi,* a species close to *T. urticae*, the behaviors that underlie within plant distribution and circadian migration remain poorly understood. But this information is essential to develop a well-adapted IPM strategy.

Among the pests damaging tomato crops, the spider mites *T. evansi* and *T. urticae* are considered as key pests in sub-Saharan Africa [18]. Indeed *T. evansi* was recently reported as a new invasive species in tomato crop in Africa [19]. It has been shown that tomato infestation by *T. evansi* can causes severe damage. This mite is characterized by a high reproductive capacity, which leads to high population levels in a short time, causing important economic damage and yield losses close to 90% [20], [21], [22], [23]. The control of *T. urticae* and *T*. *evansi* is done mainly with application of synthetic pesticides. Despite its relative efficiency, chemical control has several negative impacts as the selection of resistant individuals due to the continuous use of certain active ingredients, the reduction or elimination of beneficial species, the high toxicity of products to applicators, and the presence of residues in food [24], [25]. A viable alternative to the problems arising from excessive use of synthetic pesticides is the use of methods that provide control with social and environmental safety. In the search for such methods, natural enemies are being evaluated as biological control agents of *T*. *evansi* [26], [27], [28]. Another strategy could be the use of acaricide treated nets, emerging as a new concept of mite control. This technique of mite control is effective against mite species such as *Polyphagotarsonemus latus (Banks)* and *T. urticae* [29]. Moreover acaricide treated net could be combined with the release of predatory mites because of no chemical residues on plant. Martin et al. [29] opined that the circadian migration of *T. urticae* up and down the plant stem [30] could be the reason for the effectiveness of the acaricide treated nets. Using acaricide treated net could be a promising avenue, but its efficiency would directly depend on *T. evansi* dispersal within and among plants.

In this regard, we studied here (i) the distribution of *T. evansi* and *T. urticae* on single tomato leaves, (ii) the within plant distribution, multiplication of mites and damage on whole plants and (iii) the within plant circadian migration of *T. evansi*.

## Materials and Methods

### 1. Plants

Tomato seeds *Solanum lycopersicum* L. var. ‘Money Maker’ from the East African Seed Company, Nairobi, Kenya, were sowed in rows in soil enriched with compost in plastic seed trays. Plants (21 days-old) were transplanted into pots (22 cm diameter) each containing a mixture of red soil plus bovine manure (3: 1) and placed on benches in a greenhouse until they were 45 days old and had at least four completely developed leaves. The plants were watered daily and each pot was nourished with 3 g calcium ammonium nitrate [CAN (26% N) from Jumbo Agrovet, Nairobi, Kenya] two weeks after transplanting. Subsequently, plants were used either for the experiments or for spider mites rearing.

### 2. Mites

Spider mites *T. evansi* used in this study were obtained from a regularly regenerated colony maintained at *icipe* (International Centre of Insect Physiology and Ecology) on potted tomato plants variety ‘Money Maker’. *Tetranychus urticae*, were obtained from Real IPM Kenya Ltd Thika and reared on bean plants. The mass cultures of *T. evansi* and *T. urticae* were maintained at a temperature of 25±1°C, 50–70% relative humidity (RH) and 12 hours photoperiod.

### 3. Dispersal behavior of the mites

The dispersal behavior, the within leave and plant distribution and circadian migration of *T. evansi* and *T. urticae* mites were assessed with three experiments conducted between June and December 2012.

#### 3.1. Within leave distribution of *T. evansi* and *T. urticae*

Each experimental unit consisted of a box (Height  =  5.5 cm and length  =  17.7 cm) in which a thin layer of cotton wool was placed. Tomato leaves with five leaflets were spread upside down on the cotton. The leaves were numbered from the apex to the base. Mites in four different starting densities (1, 10, 30 and 60 females) of either *T. urticae* or *T. evansi* were placed on the first leaflet (*i.e.* at the apex). All motile stages of *T. evansi* or *T. urticae* were counted in every two days for 15 days. The boxes were kept in the incubator where temperature was kept at 25°C. Each treatment was replicated five times.

#### 3.2. Within plant distribution, multiplication of mites and damage

Tomato plants (45 days-old) with at least four completely developed leaves were infested with 100 ♀ and 10 ♂ of *T. evansi* or *T. urticae* deposited on the lowest leaf of the plant. These were maintained in the laboratory for 21 days and adult mites were counted every three days on each leaf numbered from bottom to the top. In order to estimate the degree of damage to the plant, the mean leaf damage index was ranked on a scale from 0 (no damage) to 5 (the leaf begins to shrivel) following the method described by Hussey and Scopes [31]. Each treatment was replicated six times.

#### 3.3. Within plant circadian migration activity of *T. evansi*

Migration activities of mites were recorded by video-tracking system from Noldus Information Technology, Wageningen, Netherlands. Tomato plants of 12 cm high were used and a strip of black paper (1 by 20 cm) was pasted with glue and attached to the stalk with thin wire. The tomato plants were infested at the bottom leaves with 100 mites (T_0_) and kept in the laboratory for 6 days. Tracking was carried out at T0 + 3 and T0 + 6 days. During each observation day, the migratory behavior was monitored over 24 hours and mites ascending and descending the strip of black paper were recorded later with video-tracking system. The experiment was replicated three times.

### 4. Data analyses

We used two distinct variables to analyse dispersal on single leaves. First, we created a Composite Dispersal Index (CDI) summarizing mite position on the leaf. This index allowed analysis of mite movements among leaflets. To calculate this index we assigned the value of 1 to mites found on the first leaflet, value of 2 to mites in the leaflet 2 and 3 which originated the node next to the first leaf, and value of 3 to mites in the leaflets 4 and 5 located on the next node. The formula used to calculate the index is as follows (Equation 1):
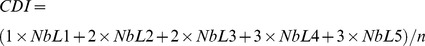



Where Nb.Lx represents number of mites on the x^th^ leaflet and *n* the total of individuals. Greater *CDI* values therefore reflect greater dispersal away from the inoculated leaflet and towards distant leaflets. Second, we also calculated the proportion of mites still present on the first leaflet, where inoculation had occurred.

For these analyses we used mixed-models, in which fixed factors were “mite species” (a discreet factor), “initial density” (a continuous factor ranging from 1 to 60 mites) and “day post inoculation” (a continuous factor ranging from 1 to 15) and all their interactions. We also included “total mite number” (a fixed continuous factor) so as to control for differential population densities among species and replicates. We further included a random, discreet factor “leaf identity” that controlled for pseudo-replication by taking into account the multiple observations on the same leaves.

On whole plants we analysed the total number of mites, as well as an index of mite distribution among leaves. This index was similar to the one used for among leaflets movements at the exception that mites received a score of 1 when on the first leaf (i.e. inoculated leaf at the bottom of the plant), 2 when on the second leaf, and so on until the 6^th^ leaf (above leaves could not be included in the analysis as they were not present in all plants at all times). Total mite number was log-transformed before analysis because *T. evansi* and *T. urticae* populations grow exponentially. This trait was analysed using an ANCOVA where each point was the average number of mites per time point (i.e. day) and mite species. Factors were “mite species”, “day post inoculation” and their interaction. To analyse the index of mite distribution within the whole tomato plant we employed the same type of mixed-model as for the index of within leaf distribution (see above). Initial fixed factors were “mite species” (a discreet factor), “total mite number on the plant” (a continuous factor) and “day post inoculation” (a continuous factor ranging from 0 to 21) and all their interactions. This model also contained the random factor “plant identity” so as to control for pseudo-replication. All above analyses were carried out with the software JMP v.10.0.2 (2012).

Mite circadian migration were analysed with the software from Noldus Technology, Wageningen, Netherlands, using the sum of mites ascending and descending the black paper strip.

## Results

### 1. Within leave distribution of *T. evansi* and *T. urticae*

**Figure 1:**
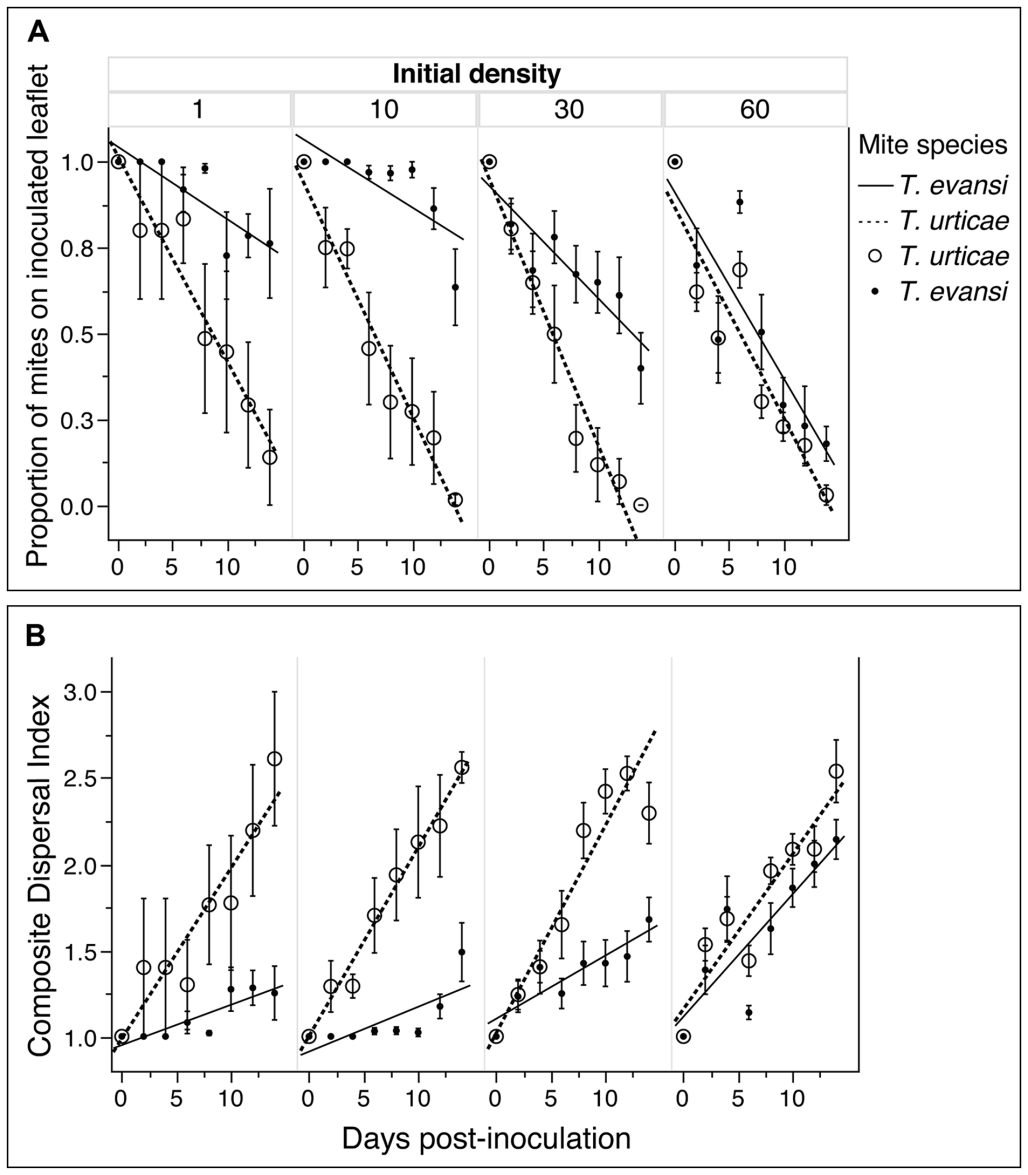
Within leave distribution of *T. evansi* and *T. urticae.* (A) Proportion of *Tetranychus evansi* and *T. urticae* on inoculated leaflet over 15 days relative to the initial density; (B) Composite Dispersal Index summarizing mites position on the leaf relative to the initial density. The error bars indicate the standard errors.

The analysis of mite species distribution on single leaves revealed different effects of initial density on *T. evansi* and *T. urticae* movements (Table 1, Figure 1). There were indeed significant interactions between “Initial density” and “Mite species” and between “Initial density”, “Mite species” and “Day post-inoculation” for both the proportion of mites that remained on the inoculated leaflet and the CDI (Figures 1a and 1b). The tendency of *T. urticae* to move away from the inoculated leaflet (Figure 1a) and colonize distant leaflets (Figure 1b) appeared independent of initial mite density, whereas these traits increased with *T. evansi* initial density. At initial density 1, 10 and 30 mites, further analyses revealed significant differences between *T. urticae* and *T. evansi* (all interactions “Mite species” x “Day post-inoculation” had P<0.001). However, at initial density 60 the interaction “Mite species” x “Day post-inoculation” was neither significant for the proportion of mites on the inoculated leaflet nor for the CDI (P>0.1).

**Table 1:** Analysis of mite position on single tomato leaves.

*Proportion of mites on inoculated leaflet*			
Factor	d.f.	F	p
Mite species	1, 36	33.1	<.0001
Initial density	1, 45	23.0	<.0001
Day post-inoculation	1, 270	449	<.0001
Mite species*Initial density	1, 36	5.46	0.0250
Mite species*Day post-inoculation	1, 269	49	<.0001
Initial density*Day post-inoculation	1, 270	12.5	0.0005
Mite species*Initial density*Day post-inoculation	1, 269	10.3	0.0015
Total mite number	1, 289	5.35	0.0214
*Leaf identity (Random factor)*	35% variance explained		

*Composite Dispersal Index*			
Factor	d.f.	F	p
Mite species	1, 36	31.1	<.0001
Initial density	1, 43	13.3	0.0007
Day post-inoculation	1, 269	407	<.0001
Mite species*Initial density	1, 36	4.80	0.0350
Mite species*Day post-inoculation	1, 269	74.1	<.0001
Initial density*Day post-inoculation	1, 270	7.57	0.0063
Mite species*Initial density*Day post-inoculation	1, 268	12.3	0.0005
Total mite number	1, 286	6.85	0.0093
*Leaf identity (Random factor)*	41% variance explained		

Mites were initially deposited on the terminal leaflet and their position tracked for 15 days. The Composite Dispersal Index (CDI) summarizes mite position on the leaf; see main text (Eq. 1) for details.

### 2. Within plant distribution, multiplication of mites and damage

**Figure 2:**
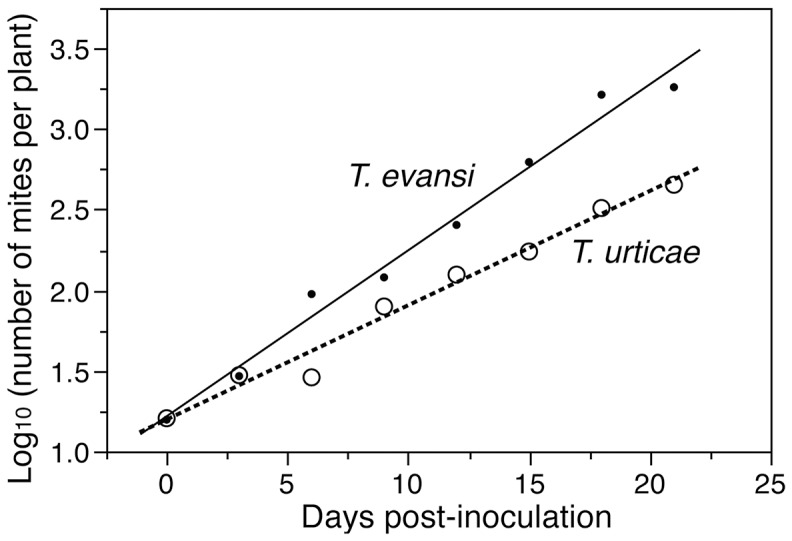
Within plant multiplication of mites. Number of *Tetranychus evansi* and *T. urticae* per tomato plant (Log10) after an initial infestation with 100 females and 10 males on the lowest leaf of the plant.

The analysis of mite numbers showed a greater multiplication of *T. evansi* than *T. urticae* (Figure 2); as revealed by the significant interaction between “Mite species” and “Day post-inoculation” (F_1,12_  =  22.1, *P* = 0.0005).

**Figure 3:**
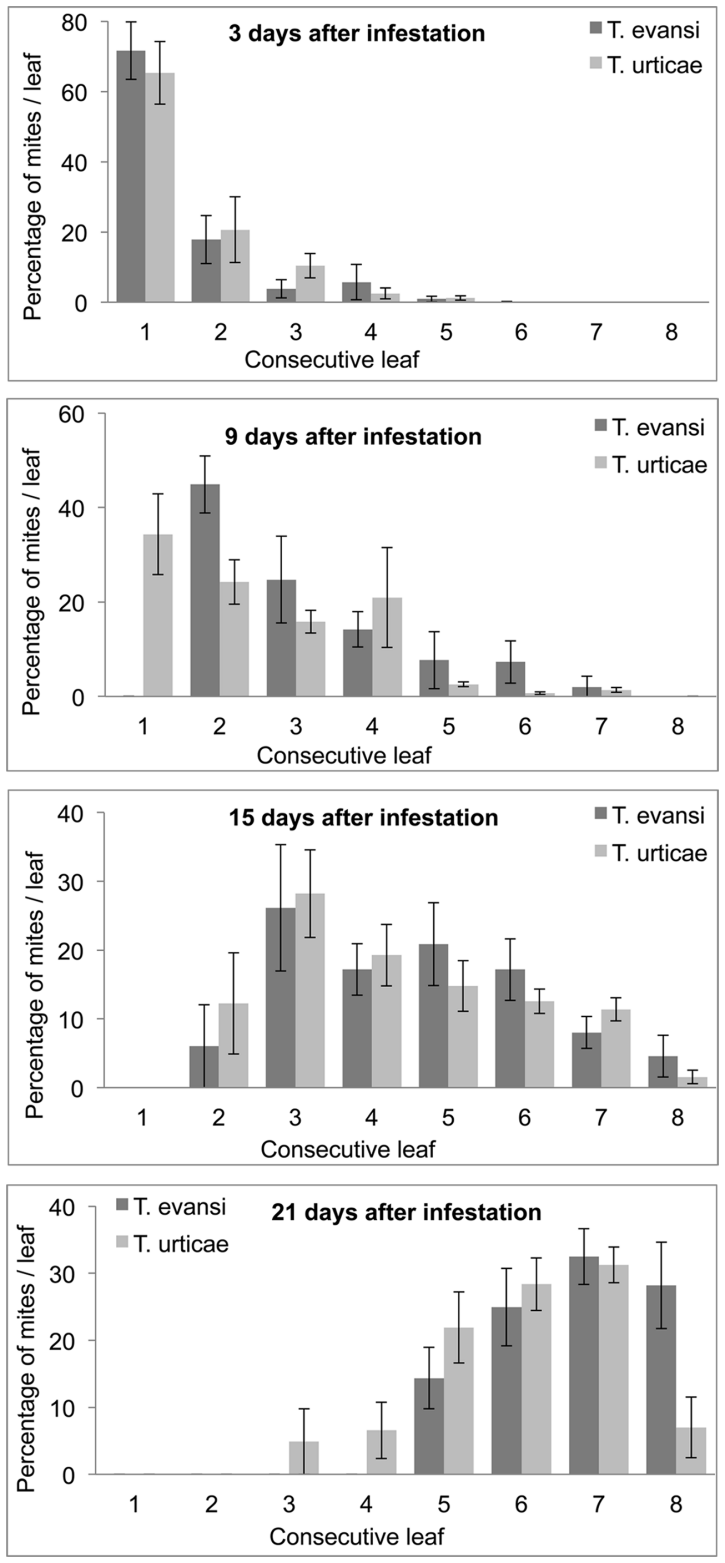
Within plant distribution of *Tetranychus evansi* and *T. urticae* motile stages on tomato plant. Average on 6 plants after 3, 9, 15 and 21 days of initial infestation (Error bars indicate the standard errors).

The within plant distribution of *T. evansi* and *T. urticae* was broadly similar over the course of our experiment (Figure 3). The analysis of the dispersal index (see methods) indicated a greater tendency of *T. evansi* than *T. urticae* to colonise the top of the plant (Interaction “Mite species” * “Days post inoculation” F_1,78_  =  6.89, *P* = 0.01). But this difference was only marginal as the slope of the regression of the index on “Days post inoculation” was 0.203 for *T. urticae* and 0.230 for *T. evansi*.

**Figure 4:**
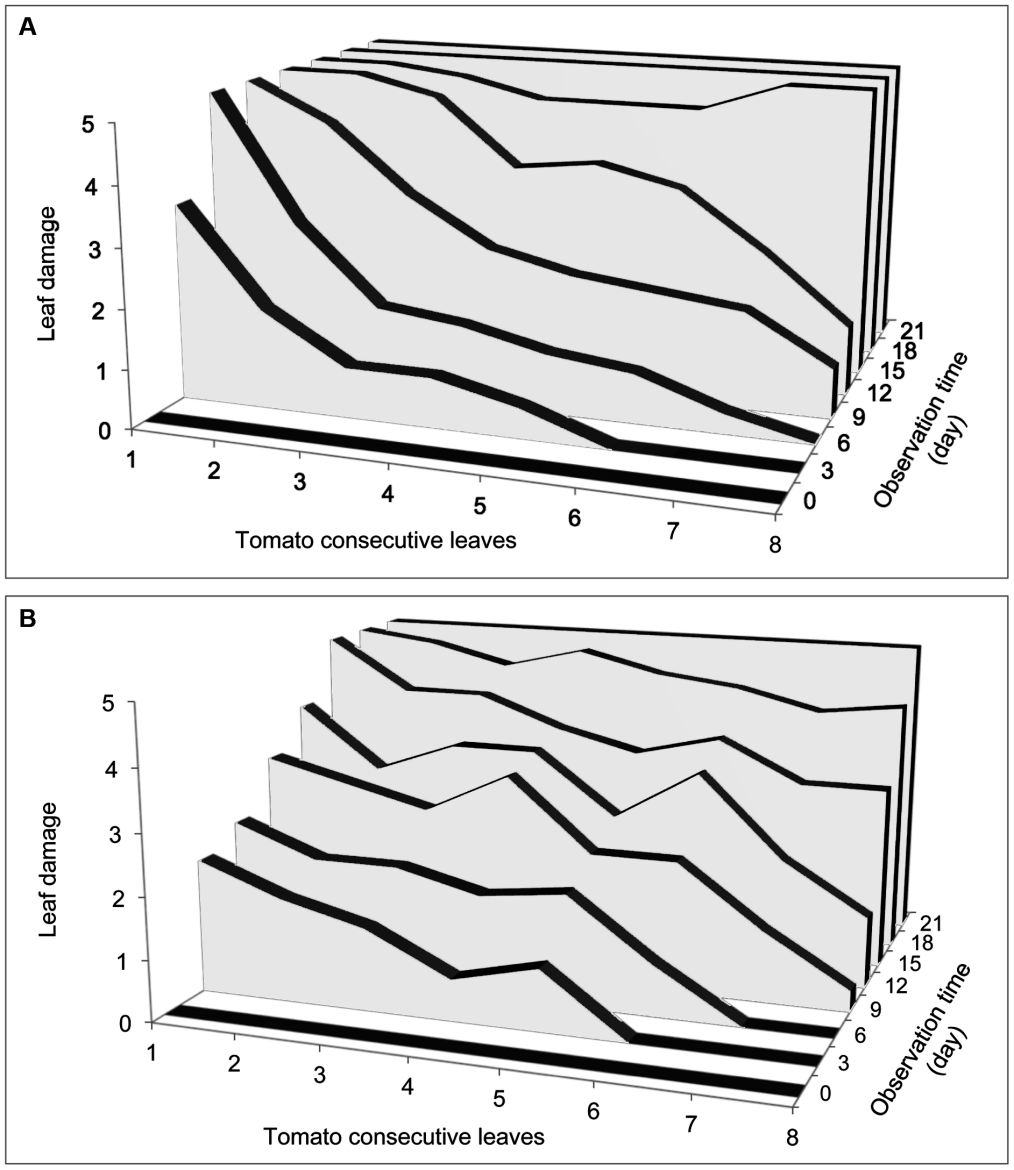
Damages by *Tetranychus evansi* and *T. urticae* on tomato plant. Leaf mean damage indices for *T. evansi* (A) and *T. urticae* (B) on tomato plant after an initial infestation with 100 females and 10 males at the bottom

Damages by *T. evansi* and *T. urticae* on tomato plant also differed (Figure 4). From the 6^th^ day, we noted severe damage (index 5) of the first leaf by *T. evansi* and by the 15^th^ day all leaves were dead (Figure 4a). However with *T. urticae* the damages were more moderate (index between 2 and 3) and evenly distributed among leaves from the 3^th^ to the 9^th^ day. Severe damage on the first leaves was observed from the 15^th^ day and it was only by the 21^st^ day the damage was severe with the index 5 for all leaves on the plant (Figure 4b).

### 3. Within plant circadian migration activity of *T. evansi*

**Figure 5:**
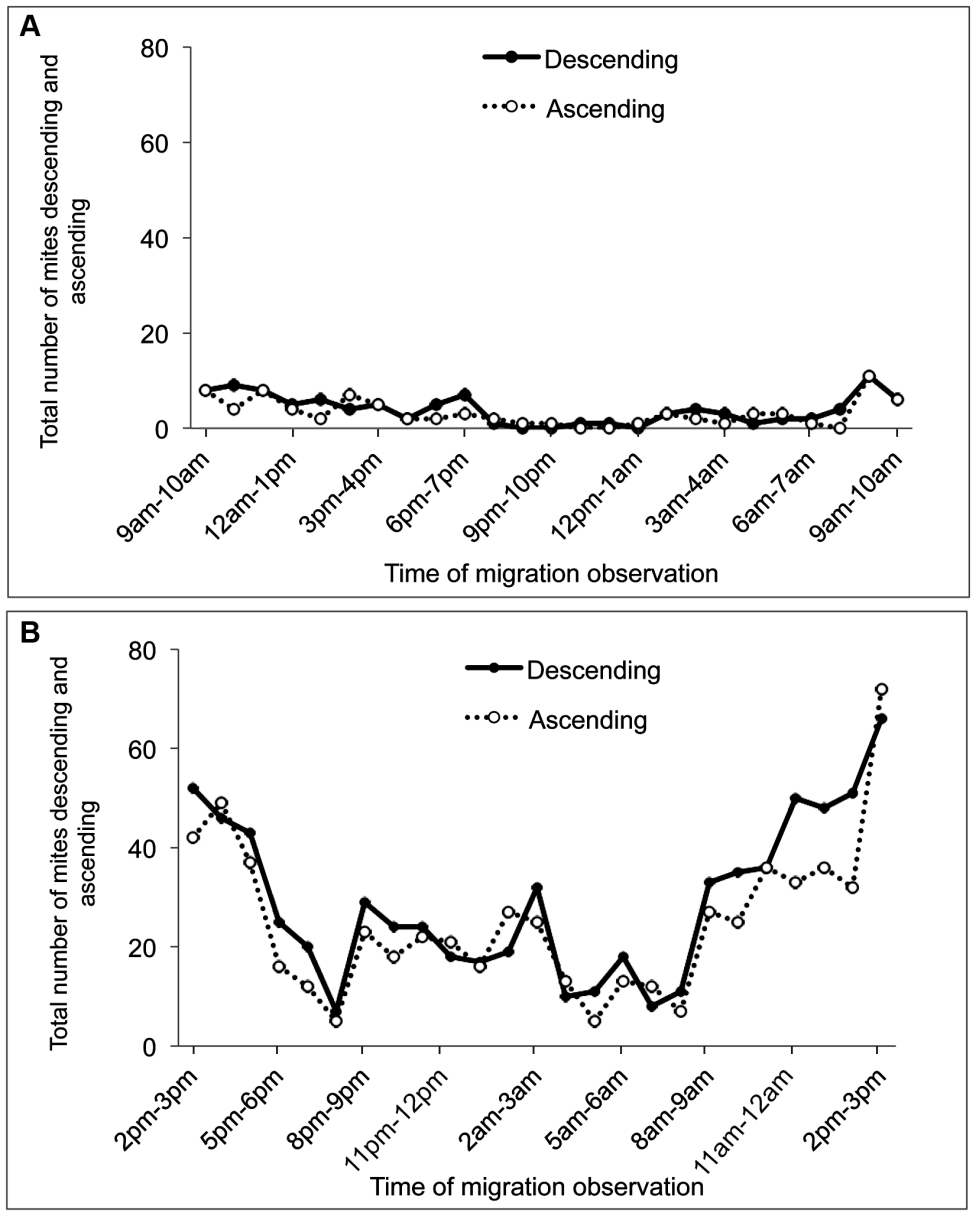
Within plant circadian migration activity of *Tetranychus evansi*. Migration activity of *T. evansi* on the tomato plant at T_0_+3 days (A) and T_0_+6 days (B). 100 females were introduced at T_0_ and each individual ascending or descending the black paper placed at the top of the plant was recorded by video tracking during 24 hours. In the figure, the number of individuals was cumulated at each observation time

The migration activity is characterised by the ascending and descending of the mites on the black paper strip on the top of tomato plant. Three days after initial infestation (T0+3), the number of motile stage per plant was quiet low (significantly 100 mites). The number of ascending and descending mites also did not differ significantly over observation times of the day indicating a very low flux of migration (Figure 5a). The total number of mites ascending over 24 h (79, Confidence interval (CI): 59–99) was similar to the total number of mites descending (96.7, CI: 74–119). The migration observed occurred during the day. Six days after initial infestation (T0+6), the number of motile stages observed on the tomato plant was 522 on average. At this high density, we observed increasing flux of migration from 8 am to 3 pm with peaks migration between 2 and 3 pm. The migration declined after 3 pm until 8 pm, later it increased until 3 am (Figure 5b). The total number of mites ascending during 24 hours (576, CI: 473–680) was broadly similar to the total number of mites descending (608, CI: 340–877).

## Discussion

Our results showed a more gregarious behavior of *T. evansi* than of *T. urticae* on single leaf as well as on whole tomato plant. On single leaves, *T. evansi* seemed initially gregarious and only dispersed to further leaflets when its density was high. By contrast, *T. urticae* exhibited dispersal movements at all times and even at low densities and did not seem to depend on initial density (i.e. similar slopes of *T. urticae* regressions in Figure 1 over the 4 initial densities). *T. evansi* thus exhibited greater density-dependent dispersal than *T. urticae* confirming previous results [8], [32]. On whole tomato plant, *T. urticae* seems to stay longer on initial leaves than *T. evansi*. It appeared also that T. urticae is distributed on the whole plant with quite the same density. In the contrary it appeared that *T. evansi* dispersed mainly after the destruction of food shortage of lower leaves. *Tetranychus evansi'*s population grew exponentially and destroyed the tomato plant in 15 days while *T. urticae* caused moderate damage on tomato leaves due to its distribution on the plant and total plant destruction was recorded 21 days after initial infestation. Our result confirms previous studies showing higher reproductive rates of *T. evansi* compared to *T. urticae* on tomato plants [20], [21], [23]. The high distribution of *T. urticae* on whole tomato plant, evidenced by the moderate damage on leaves and its low population growth rate compared to *T. evansi* could explain a better resilience of tomato plant to *T. urticae* compared with *T. evansi* infestations. This gregarious behavior of *T. evansi* combined with its rapid population growth rate, may explain why tomato plants can be severely damaged by *T. evansi* infestations and how suddenly a tomato crop can be highly infested by them. The dispersal behavior of *T. evansi* makes it easy the localisation of infested tomato plants appearing highly damaged. The early control of *T. evansi* on these high spots could reduce the risk of outbreaks in the crop. The different dispersal behavior of *T. evansi* and *T. urticae* (density-dependent and density-independent, respectively) indicate these two species are not exposed to the same stresses and have different dynamics. *T. evansi* has to face food shortages and host plant desiccation for dispersal [33], [34]. But *T. urticae* females disperse by walking to new leaves even at low densities when food is not scarce [35]. Yano [36] reported that females of *T. urticae* can disperse alone and start new colonies, after which sib-mating occurs among their offspring. In both species, when populations grow to the extent that individuals face both food limitations and host plant desiccation mites often build dense silk webs [35], [36] further aggregate to form silk balls involved in long-range dispersal and the colonisation of distant plants [37], [38].

In our study we showed ascending and descending movement by *T. evansi* as already observed for *T. urticae* [16]. Such circadian migration was even observed at low population density, but was less intense than at high population density. In both cases, the total number of *T. evansi* ascending was similar to the total number of *T. evansi* descending. After the exhaustion of food shortage we observed that the mites aggregated at the top of the plant to form silk balls as observed with *T. urticae* [23]. It is however unknown whether circadian migration was due to a few mites migrating many times or a high number of mites migrating few times. Further studies would be necessary to understand what proportion of the mites is actually involved in circadian migration and how far this migration takes place within a plant. If all or almost all mites undergo circadian migration, most mites could enter in contact with an acaricide net covering the plant even at low density [29]. Circadian migration would therefore accentuate the efficiency of a pest control strategy based on acaricide treated nets placed on hot spot. Our results on *T. evansi* and *T. urticae* dispersal on tomato plant have several implications in the use of acaricide treated net for their management. The low migration behavior of *T. evansi* at lower density could render an acaricide treated net less effective. In this case, we could combine these nets with the release of predatory mites such as *Phytoseiulus longipes* [27] to catch the mites between a rock and a hard place. The migration behavior of *T. evansi* at high density should enhance the efficiency of acaricide treated net covering the infested plant. Thus an acaricide treated net placed on a spot of infested plants could be very effective to control *T. evansi* as the mite would be confined by the netting and killed when spreading to other plants. Moreover, as the number of *T. evansi* ascending and descending appeared as quite similar to the total number of motile mites present on the plant at both low and high densities, this opens an avenue for the use of acaricide treated net on infested plants. Indeed, it suggests that many of the mites may enter into contact with the net and therefore be killed by the acaricide.

In conclusion, we found that: (1) *T. evansi* initially showed a gregarious behavior before displaying dispersal behavior (i.e. positive density-dependent dispersal) while *T. urticae* showed mainly dispersal behavior (i.e. density-independent dispersal); (2) *T. evansi* multiplied faster than *T. urticae* on tomato, causing severe damage and killing tomato plants faster than *T. urticae*; (3) *T. evansi* circadian migration peaked in the morning and in the evening and the total number of ascending and descending mites over 24 h was similar and close to overall population density. These results indicate that acaricide treated net may be efficient to limit *T. evansi* infestations on tomato crop.
